# Is the Nickel-Dependent Urease Complex of *Cryptococcus* the Pathogen’s Achilles’ Heel?

**DOI:** 10.1128/mBio.00408-13

**Published:** 2013-06-25

**Authors:** Carl A. Morrow, James A. Fraser

**Affiliations:** Australian Infectious Diseases Research Centre, School of Chemistry and Molecular Biosciences, the University of Queensland, St. Lucia, Brisbane, Queensland, Australia

## Abstract

The nitrogen-scavenging enzyme urease has been coopted in a variety of pathogenic organisms as a virulence factor, most notoriously to neutralize stomach acid and establish infection by the gastric pathogen *Helicobacter pylori*. The opportunistic fungal pathogen *Cryptococcus neoformans* also utilizes urease as a virulence factor, only in this case to invade the central nervous system (CNS) via the blood-brain barrier and cause life-threatening meningoencephalitis. A recent study [A. Singh, R. Panting, A. Varma, T. Saijo, K. Waldron, A. Jong, P. Ngamskulrungroj, Y. Chan, J. Rutherford, K. Kwon-Chung, mBio 4(3):e00220-13] genetically and biochemically characterizes the accessory proteins required for successful activation of the urease protein complex, including the essential nickel cofactor. The accessory proteins Ure4, Ure6, and Ure7 are all essential for urease function. Ure7 appears to combine the roles of two bacterial accessory proteins: it incorporates both the GTPase activity and nickel chaperone properties of UreE, a bacterial protein whose homolog is missing in the fungi. An accompanying nickel transporter, Nic1, is responsible for most, but not all, nickel uptake into the fungal cell. Mutants of the core urease protein Ure1, accessory protein Ure7, and transporter Nic1 are all attenuated for invasion of the CNS of mice, and urease activity may directly affect integrity of the tight junction of the endothelial cells of the blood-brain barrier, the network of proteins that limits paracellular permeability. This work highlights the potential of urease, its accessory proteins, and nickel transport as potential chemotherapeutic targets.

## Commentary

Urea is a nitrogenous compound that plays important roles in nitrogen metabolism and renal function and is frequently excreted as a waste product by mammals. Widely used as a fertilizer, many plants possess the metalloenzyme urease to break down urea into ammonia for use as a nitrogen source, as do microbes. Urease in bacteria is generally a heterotrimer comprised of the proteins UreA, UreB, and UreC, with a binuclear nickel (Ni^2+^) center. The accessory proteins UreD, UreE, UreF, and UreG are required for assembly of the functional apoenzyme complex. In addition, high-affinity nickel transporters are required to mediate metal ion uptake from the environment. Plant and fungal ureases are homotrimers or homohexamers with nickel centers that require only homologs of the three accessory proteins UreD, UreF, and UreG for function (1).

Beyond its role in nitrogen scavenging, urease activity is an important virulence factor in a number of pathogens, such as the bacterial species *Helicobacter pylori* and *Proteus mirabilis* and fungal species *Coccidioides immitis* and *Cryptococcus neoformans*. Urease has been especially well characterized in the gastric ulcer-causing *H. pylori*, which colonizes the stomach epithelium and excretes urease to break down the abundant urea into ammonia and bicarbonate, neutralizing the local pH (2). Among fungal pathogens that initiate infection via the lungs, urease serves to promote pulmonary colonization through alkalinization, local tissue damage, and an inappropriate immune response in both *Coccidioides immitis* and *Coccidioides posadasii* (3).

In the important pathogen *Cryptococcus neoformans*, urease promotes sequestration of the fungus within the microcapillary beds of the blood-brain barrier (BBB) and transmigration through the barrier cells to facilitate invasion of the central nervous system (CNS). *C. neoformans* is an opportunistic pathogen and the primary cause of fungal meningoencephalitis in humans, responsible for up to a million infections and roughly 600,000 deaths per year (4). The fungus predominantly infects immunocompromised individuals, such as AIDS patients and those receiving immunosuppressive medication, although the closely related sister species *Cryptococcus gattii* usually infects apparently healthy and immunocompetent hosts. These infections are enabled by several characteristic virulence factors, including a polysaccharide capsule, melanin, and a variety of extracellular proteins, including proteases, phospholipases, and urease. Urease-negative strains very rarely penetrate the CNS or cause disease, and urease is known to promote sequestration of cryptococcal cells in the microvasculature of the brain (5). Recent, excellent work from Shi et al. (6) using intravital microscopy has demonstrated for the first time *Cryptococcus* invasion of the mouse brain *in vivo* and revealed that a urease-deficient strain was severely impaired in transmigrating the BBB. Further, an inhibitor of urease, flurofamide, also severely impaired cryptococcal transmigration. While the specific role of urease protein in BBB invasion is not known, it has been suggested that the extracellular enzymatic degradation of urea to toxic ammonia may damage the endothelial cells and lead to an increase in permeability.

A recent collaborative work in *mBio* by Singh et al. (7), from the laboratories of June Kwon-Chung and Julian Rutherford, has provided insight into urease activity in *Cryptococcus* by investigating the role of the accessory proteins Ure4, Ure6, and Ure7 (homologs of UreD, UreF, and UreG, respectively), as well as the required nickel ions, in activation of the urease apoenzyme. This study represents the first time that both the enzyme and its accessory proteins have been genetically and biochemically characterized in fungi. All three accessory proteins were found to be essential for growth on urea as the sole nitrogen source and for enzyme activity in both classical Christensen’s urea agar plates and in crude cell extracts. Interaction of the accessory proteins with the urease protein Ure1 and with each other was also confirmed using a yeast two-hybrid assay.

All known *Basidiomycota*, *Zygomycota*, and *Ascomycota* are urease positive, while the *Saccharomycetes* and a large number of other higher-classified fungi lack urease, its accessory proteins, and attendant nickel transporter. In these yeasts, such as *Saccharomyces cerevisiae* and *Candida albicans*, urea is instead metabolized by a urea amidolyase (Dur1,2), a biotin-requiring enzyme that contains both urea carboxylase and allophanate hydrolase activities to convert urea, ATP, and bicarbonate to ammonia and carbon dioxide (8).

All but one urease studied so far require nickel as a cofactor for enzymatic activity (9), and Singh et al. used inductively coupled plasma mass spectrometry (ICP-MS) to confirm the presence of nickel in urease-positive soluble *C. neoformans* cell extract fractions. Incorporation of nickel into the enzyme was also demonstrated to be dependent on the presence of the Ure7 accessory protein through similar assays in a ∆*ure7* mutant. Interestingly, the Ure7 accessory protein appears to combine the functions of two of the bacterial accessory proteins. It is a homolog of GTPase UreG, but cryptococcal Ure7 also includes an additional N-terminal histidine-containing region that appears to bind nickel, apparently replacing the nickel-binding activity of the missing bacterial UreE homolog (a nickel chaperone). Accordingly, mutagenesis of key histidine residues reduced or completely abolished both the ability of Ure7 to bind nickel and the urease activity of Ure1. The nickel transporter homolog Nic1 was subsequently deleted and demonstrated to be required for urease activity, a defect that could be overcome if excess exogenous nickel was added to the medium.

Crucially, accessory protein ∆*ure7* and nickel transporter ∆*nic1* mutants both displayed greatly attenuated virulence, as determined by brain CFU after 24 h of infection. CFU in the brain were comparable to those of the ∆*ure1* strain, demonstrating that urease activity and not the urease protein *per se* was responsible for impaired pathogenicity. Urease-deficient cells are still able to penetrate the blood-brain barrier, however, just at a much reduced rate; classic studies by Cox and colleagues demonstrated that urease activity does not affect virulence of strains when injected directly into the brain of rabbits (10). Although a lack of the urease protein directly leads to reduced cryptococcal transmigration of brain microvasculature, as penetration of the CNS is still possible in urease-negative strains but not possible in heat-killed strains, this suggests that further mechanisms that promote invasion remain to be elucidated. Recent exciting work has revealed that uptake appears to be mediated through the hyaluronic acid receptors, such as CD44 and RHAMM, and hyaluronic acid decorating the polysaccharide capsule of *C. neoformans*, and blocking this interaction via anti-CD44 antibodies, bikunin, or simvastatin can reduce fungal association with human brain microvascular endothelial cells (HBMECs) that form part of the BBB (11). This process involves membrane lipid raft-dependent endocytosis, and inhibition of dual-specificity tyrosine phosphorylation-regulated kinase 3 (DYRK3) or caveolin-1 also reduces the ability of *Cryptococcus* to invade HBMECs (12). These processes are not necessarily exclusive to urease activity. Notably, cryptococcal invasion of the CNS via the “Trojan horse” mechanism, within the phagolysosome of a macrophage, could also still occur.

Integrity of the tight junction cells was also assayed for the first time by Singh et al. (7) using urease-positive and -negative cryptococcal cells in contact with HBMECs. Levels of the tight junction protein ZO-1 were measured by Western blot analysis, which displayed a reduction in protein levels after 24 h of exposure to urease-positive *Cryptococcus*. As there was a modest reduction, it would be interesting if future work extended this analysis to other tight junction integrity markers, such as occludin and claudin-5, in HBMECs to interrogate potential changes in expression, abundance, and localization (13).

A highlight of Singh et al.’s work (7) was the compelling analysis of the role of nickel in the activity of the urease apoenzyme. Only eight nickel-dependent enzymes have been identified so far (primarily from prokaryotic sources), and although nickel depletion studies in rats have demonstrated decreased growth, altered liver development, and impaired iron absorption, no nickel-requiring metalloenzymes have been identified in vertebrates (1, 14). It follows that targeting an Ni-requiring process or enzyme may be a legitimate and specific method to target a pathogen, especially when the nickel-containing enzyme governs entry to the CNS and is a notable virulence factor, as in the case of *Cryptococcus*. Urease inhibition by compounds such as flurofamide in *C. neoformans* or acetohydroxamic acid in *C. immitis* has been demonstrated to lead to improved survival in a mouse model, reduction in CFU, or reduction of urease activity (3, 6). Here, a nickel chelator may also be a viable strategy. The study demonstrates that the ∆*nic1* nickel transporter mutant shows no detectable urease activity despite the presence of alternate nickel transporters (probably calcium channels). As very high concentrations of nickel (10 µM) are required to complement this knockout phenotype and human blood contains only ~0.5 nM, inhibition of Nic1 may also prove an effective strategy in combating *Cryptococcus* (14, 15).

There remain a number of tantalizing details to decipher concerning urease activity in fungal pathogens: how exactly does urease promote lung invasion, and in *Cryptococcus*, what is the specific mechanism by which urease enhances BBB transmigration? Is it a pH change effected through ammonia and bicarbonate, or some other role? And what influence does urease activity have on BBB penetration through the recently uncovered endocytic lipid raft uptake pathway, if any? Utility of urease or nickel uptake inhibition as viable therapeutic targets has promise; however, as ∆*ure1* strains are equally virulent in the CNS, pharmacological intervention would have to be prophylactic, such as for patients receiving an immunosuppressive medication, and may be ineffective for patients presenting with cryptococcal meningoencephalitis.
